# Immunomodulation of prostate cancer cells after low energy focused ultrasound

**DOI:** 10.1186/2050-5736-3-S1-O63

**Published:** 2015-06-30

**Authors:** Karin Skalina, Huagang Zhang, Lisa Scandiuzzi, Indranil Basu, Chandan Guha

**Affiliations:** 1 Albert Einstein College of Medicine, New York, New York, United States; 2 Montefiore Medical Center, New York, New York, United States

## Background/introduction

**Figure 1:**
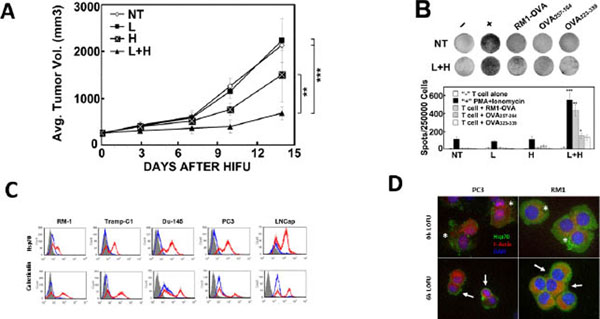
A. Tumor Growth Retardation. B. LOFU+HIFU induced tumor-specific T cell response. C. Flow cytometry of HSP70 and calreticulin of LOFU-treated prostate cancer cells. D. IF of RM-1 and PC3 cells stained for HSP70

High-Intensity Focused Ultrasound (HIFU) is a promising non-invasive treatment for localized solid tumors. While patients treated with HIFU show symptomatic improvement, the majority die from local recurrence and metastases due to incomplete tumor ablation and inability to control spread outside the primary tumor site. Therefore, improved local control and a concomitant systemic therapeutic effect would be valuable for successful tumor control by HIFU. Low intensity focused ultrasound (LOFU) induces sonic stress by raising the temperature without killing the cells. We demonstrated that tumor pre-treatment with LOFU prior to HIFU results in tumor growth retardation (Figure 1A) and induces a Th1 predominant immune response (Figure 1B). To further validate the immunological consequences of LOFU pre-treatment, we investigated the immunomodulation of prostate cancer caused by LOFU. We previously demonstrated that LOFU induced expression of genes related to the unfolded protein response (UPR) and endoplasmic reticulum (ER) stress. We hypothesize that LOFU increases immunomodulatory surface signals, such as heat shock protein 70 (HSP70) and calreticulin. HSP70 is an endogenous “danger” signal, which can activate dendritic cells. Calreticulin is an “eat me” signal, which encourages phagocytosis and thus antigen processing for presentation on cell surface MHC for T cell recognition. Thus, sequential administration of LOFU and HIFU provides a source of tumor antigens and endogenous “danger” signals for dendritic cell (DC) activation, thereby inducing a tumor-specific systemic immune response that augments the efficacy of therapeutic ultrasound to control both local and systemic disease.

## Methods

LOFU treatment was performed on the Philips Therapy and Imaging Probe System (TIPS, Philips Research Briarcliff, USA) using 3W, 100% duty cycle, 1.5 seconds, 1 mm spacing. For IF analysis, LOFU-treated cells were cytospined and fixed with 4% paraformaldehyde and stained with rabbit ant-HSP70 overnight, followed by incubation with a secondary goat anti-rabbit PE conjugated and anti-phalloidin antibodies for 1 hour at room temperature. DAPI was included in the mounting medium and slides were analyzed using an Inverted Olympus IX81. Flow cytometry staining included a live/dead cell marker to isolate only living cells expressing HSP70 on the cell surface.

## Results and conclusions

The immunomodulatory effect of LOFU was analyzed on prostate cancer cell lines, both human and murine, by detecting the surface expression of HSP70 and calreticulin by flow cytometry & immunofluorescence (IF) six hours following LOFU treatment of a cell pellet. LOFU significantly induced cell surface HSP70 expression and calreticulin *in vitro* in human and mouse prostate cancer cell lines, as shown by flow cytometry (Figure 1C) and confirmed by IF (Figure 1D). Surface HSP70 is a danger signal which can activate dendritic cells to induce an anti-tumor immune response. Surface calreticulin acts as a “eat me” signal for phagocytic cells, resulting in increased phagocytosis and antigen processing. Immunomodulation by LOFU can attract DCs to the tumor and induce a Th1 predominant immune response.

